# Coronary CT Angiography in PCI Planning: Advances, Clinical Applications, and Challenges

**DOI:** 10.3390/jcdd13060239

**Published:** 2026-05-31

**Authors:** Ahmed Mahmoud Elsoudy, Luciano Candilio

**Affiliations:** Department of Cardiology, Royal Free Hospital, London NW3 2QG, UK; ahmed.elsoudy@nhs.net

**Keywords:** coronary computed tomography angiography, CTCA, percutaneous coronary intervention, PCI planning, FFR-CT, fractional flow reserve, artificial intelligence, chronic total occlusion, plaque characterization, interventional cardiology

## Abstract

Background: Interventional cardiology is increasingly being reshaped by rapid progress in non-invasive cardiovascular imaging. Coronary computed tomography angiography (CTCA), once used mainly to exclude obstructive coronary artery disease (CAD), is now being adopted as a broader planning instrument before percutaneous coronary intervention (PCI). Its ability to generate high-resolution three-dimensional visualization of the coronary tree, together with functional assessment through CT-derived fractional flow reserve (FFR-CT) and more advanced plaque analysis supported by artificial intelligence (AI), has expanded its relevance from diagnosis alone to strategic procedural preparation. In this setting, CTCA can help refine lesion assessment, anticipate technical complexity, and support better procedural and clinical outcomes. Technological Advancements: The value of CTCA for both diagnosis and risk stratification has increased substantially with recent technical innovation. Among the most important developments is the maturation of FFR-CT, which enables non-invasive physiological interrogation of coronary stenoses using computational modeling. At the same time, artificial intelligence and deep learning tools are reshaping the CTCA workflow by improving automation, facilitating plaque analysis, and highlighting adverse plaque characteristics such as positive remodeling, spotty calcification, and the napkin-ring sign. Clinical Applications: In modern catheterization practice, CTCA is increasingly used to address anatomically demanding scenarios. Its role is particularly valuable in chronic total occlusion (CTO) intervention, where it can delineate occlusion length, stump characteristics, vessel course, and collateral anatomy before the procedure. Its usefulness also extends beyond CTO PCI by supporting vessel sizing, stent planning, and anticipation of lesion preparation requirements in complex coronary disease. Challenges: Despite these advantages, several barriers continue to limit wider implementation, including blooming from heavy calcification, radiation burden, contrast-related renal concerns, and the practical difficulty of embedding CTCA-based planning into routine workflows. Conclusions: CTCA is becoming an increasingly important adjunct in PCI planning because it can combine anatomical definition, physiological interpretation, and plaque-level information before invasive treatment is undertaken. Overall, this review emphasizes CTCA not only as a diagnostic modality, but also as a practical pre-procedural roadmap that can guide lesion selection, stent planning, calcium modification strategies, and overall PCI strategy.

## 1. Introduction

ICA provides only a Luminogram—a silhouette of the contrast-filled lumen—offering no information about plaque composition, vessel wall morphology, or the hemodynamic significance of intermediate stenoses [1]. It is inherently two-dimensional in a three-dimensional anatomical world, and inter-observer variability in stenosis grading remains a persistent concern [2]. These shortcomings become particularly consequential in complex anatomical scenarios, where a mischaracterized lesion can translate directly into a suboptimal procedural strategy. The emergence of coronary computed tomography angiography as a pre-procedural planning tool represents one of the most clinically significant developments in modern cardiology. Early CTCA systems, constrained by limited temporal resolution and high radiation doses, were largely confined to ruling out obstructive CAD in low-to-intermediate risk patients. The introduction of 64-slice CT scanners in the mid-2000s marked a turning point, enabling reliable visualization of the entire coronary tree in a single breath-hold [3]. Subsequent hardware advances—dual-source CT, wide-detector arrays, prospective ECG-gating, and most recently photon-counting detector CT—have progressively expanded the diagnostic envelope, enabling CTCA to deliver isotropic three-dimensional reconstructions with sub-millimeter spatial resolution [4]. Crucially, the clinical utility of CTCA has been validated in large-scale randomized trials. The SCOT-HEART trial [5], enrolling 4146 patients with stable chest pain across 12 Scottish centers, demonstrated that a CTCA-guided diagnostic strategy not only clarified the etiology of chest pain more accurately than standard care but also led to a 41% relative reduction in coronary heart disease death or non-fatal myocardial infarction at 10-year follow-up—a finding reported in *The Lancet* in 2025 [5]. The PROMISE trial [6], with 10,003 participants randomized to CTCA versus functional testing, established that CTCA was non-inferior for clinical outcomes while substantially reducing the rate of catheterization without obstructive CAD [6]. These landmark datasets have fundamentally reshaped clinical guidelines, with CTCA now carrying a Class I recommendation as the first-line investigation for stable chest pain in both ESC and NICE guidelines [7,8]. What this review addresses, however, is a dimension of CTCA utility that has received comparatively less systematic attention: its role not merely in diagnosis, but in the active planning and optimization of PCI. The integration of FFR-CT, AI-driven plaque analysis, and virtual PCI simulation into the CTCA workflow has created a genuinely new paradigm—one in which the interventional cardiologist arrives in the catheterization laboratory armed with a comprehensive anatomical and physiological roadmap of the patient’s coronary tree. This review synthesizes the current evidence base, critically appraises the key clinical trials, and outlines the trajectory of this rapidly evolving field.

### 1.1. The Historical Arc: From Plain Radiography to CTCA-Based PCI Planning

The evolution of coronary imaging has progressively reduced anatomical uncertainty and improved procedural decision-making. For decades, invasive coronary angiography remained the reference standard because it provided real-time visualization of the coronary lumen. However, its fundamental limitation persisted: it is a two-dimensional luminographic technique that provides limited information about vessel wall morphology, plaque composition, lesion length, calcification burden, and the true three-dimensional course of complex coronary anatomy. Non-invasive coronary imaging entered a new era with the development of electron beam CT and, subsequently, multi-detector CT. The advent of 64-slice CT scanners from 2004 onwards enabled reliable non-invasive visualization of the coronary arteries [9]. Over the following two decades, advances in scanner hardware and image reconstruction, including spectral CT, AI-based reconstruction, and photon-counting detector CT, progressively improved spatial resolution, reduced artefacts, and lowered radiation exposure [10]. As a result, CTCA has evolved from a diagnostic tool for detecting or excluding obstructive CAD into a potential pre-procedural planning platform capable of supporting PCI strategy through three-dimensional anatomical mapping, plaque assessment, and integration with functional and AI-based analysis.

### 1.2. Limitations of Invasive Coronary Angiography as a Sole Planning Tool

Despite its central role in coronary assessment, invasive coronary angiography (ICA) has several important limitations when used as a standalone tool for PCI planning. The most fundamental limitation is its two-dimensional representation of a three-dimensional structure, which may lead to foreshortening, vessel overlap, and inaccurate estimation of lesion length and spatial orientation [11]. In addition, ICA provides only a Luminogram and does not allow direct assessment of plaque composition or vessel wall morphology. As a result, lesions with similar angiographic severity may have markedly different underlying pathology and procedural implications [12]. This limitation becomes particularly relevant in complex PCI, where knowledge of plaque characteristics—such as calcification, lipid-rich content, or fibrous structure—can influence lesion preparation strategy and device selection. The physiological significance of intermediate coronary stenoses also cannot be reliably determined by angiography alone. Landmark studies such as the DEFER and FAME trials demonstrated that fractional flow reserve (FFR)-guided PCI improves clinical outcomes compared with angiography-guided strategies [13,14]. However, invasive FFR measurement requires additional procedural steps, including pressure-wire manipulation and pharmacological hyperemia, increasing procedural time, complexity, and cost. These limitations highlight the need for complementary imaging modalities that provide integrated anatomical and functional information before intervention. In this context, CTCA—particularly when combined with FFR-CT and advanced plaque analysis—offers the potential to overcome several of these constraints and to support more accurate, pre-procedural PCI planning.

### 1.3. CTCA as a Pre-Procedural Planning Platform: The Emerging Paradigm

The role of coronary computed tomography angiography (CTCA) has evolved significantly from a diagnostic tool for detecting or excluding coronary artery disease to a comprehensive platform for pre-procedural planning. This transition reflects a broader shift in interventional cardiology toward precision-guided interventions, where detailed anatomical and functional information is used to optimize procedural strategy before entering the catheterization laboratory [15]. In practical terms, CTCA-guided PCI planning provides a three-dimensional roadmap of the coronary tree, enabling accurate assessment of vessel course, lesion length, tortuosity, and spatial relationships between target lesions and adjacent structures [16]. Beyond anatomical visualization, CTCA allows non-invasive functional assessment through CT-derived fractional flow reserve (FFR-CT), which can identify hemodynamically significant lesions and guide revascularization decisions without the need for invasive pressure-wire measurements [17]. In addition, advanced plaque characterization—including identification of positive remodeling, low-attenuation plaque, and spotty calcification—provides insight into lesion vulnerability and procedural complexity [18]. From an interventional perspective, these CTCA-derived parameters can be directly translated into procedural decisions. Lesion length and vessel tortuosity may guide guidewire and catheter selection, while calcium distribution may determine the need for lesion preparation techniques such as rotational atherectomy or intravascular lithotripsy. Furthermore, accurate measurement of proximal and distal reference vessel diameters can support stent sizing and landing-zone selection, potentially reducing complications such as stent underexpansion or geographic miss. In selected cases, CTCA-based virtual PCI simulation has also emerged as a novel approach to predict post-procedural physiological outcomes. By modeling stent deployment and recalculating FFR values, this technique may allow optimization of PCI strategy before intervention is performed [19]. The clinical relevance of this approach is supported by a growing body of evidence. Emerging studies specifically evaluating CTCA-guided PCI planning have demonstrated that pre-procedural CT assessment can improve lesion characterization, facilitate procedural strategy selection, optimize stent sizing, and predict the need for calcium-modification techniques before intervention [19]. Together, these developments support a paradigm shift in which CTCA is not only used to decide whether to perform PCI, but also to determine how the procedure should be performed, enabling more informed, efficient, and personalized interventional strategies.

## 2. Technological Advances: FFR-CT, Artificial Intelligence, and Next-Generation Hardware

The transformation of CTCA from a diagnostic exclusion tool into a comprehensive PCI planning platform has been driven by three converging technological advances: the development and clinical validation of FFR-CT, the integration of artificial intelligence into image acquisition and analysis workflows, and the emergence of next-generation CT hardware. Each of these developments deserves detailed examination.

### 2.1. CT-Derived Fractional Flow Reserve (FFR-CT)

**Figure 1:**
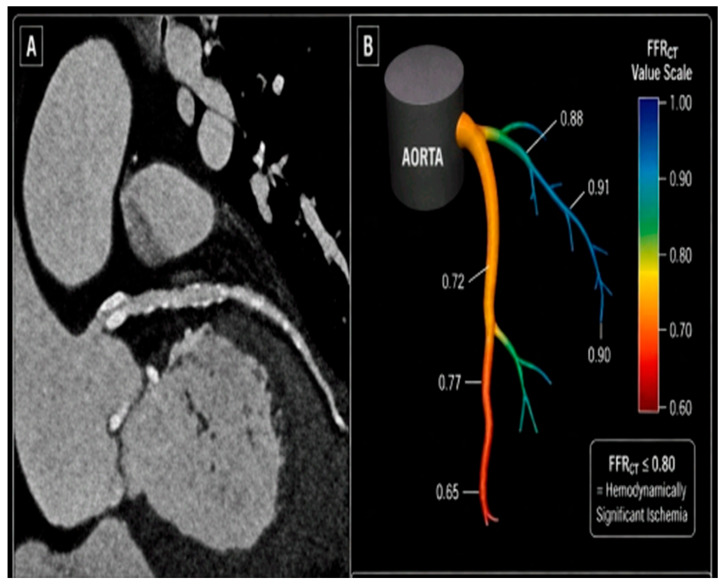
CTCA-guided PCI for significant LM stenosis. (**A**) CTCA reconstruction demonstrating severe disease involving the LM-LAD axis. (**B**) FFR-CT physiological map demonstrating lesion-specific pressure reduction and ischemia assessment across the coronary tree. All schematic illustrations were created by the authors.

Fractional flow reserve (FFR) is well established as the gold standard for the physiological assessment of coronary stenoses, allowing differentiation between lesions that are hemodynamically significant and those that can be safely deferred [20]. Invasive FFR measurement requires a pressure-sensing guidewire and pharmacological induction of hyperemia, which increases procedural time, complexity, and cost. CT-derived fractional flow reserve (FFR-CT) replicates this physiological assessment non-invasively by applying computational fluid dynamics (CFD) to standard CTCA datasets, generating a three-dimensional model of coronary blood flow and pressure gradients [21,22]. This results in a colour-coded map of coronary physiology, with values ≤ 0.80 indicating flow-limiting lesions that may benefit from revascularisation. An example of FFR-CT-based physiological mapping is illustrated in Figure 1.

From a PCI planning perspective, FFR-CT provides lesion-specific physiological information before the procedure, enabling identification of functionally significant lesions and helping to avoid unnecessary stenting of non-ischemic lesions.

This is particularly valuable in multivessel disease, where anatomical severity alone may not reflect true functional significance.

The clinical validity of FFR-CT has been demonstrated in several key studies. The DISCOVER-FLOW trial [23] was the first prospective multicenter study comparing FFR-CT with invasive FFR, demonstrating improved diagnostic performance over CTCA alone [24]. Subsequent trials, including DeFACTO and NXT, further validated the accuracy and reliability of FFR-CT in detecting haemodynamically significant stenoses [25]. Importantly, the PLATFORM trial showed that an FFR-CT-guided strategy significantly reduced unnecessary invasive angiography without compromising clinical outcomes [26]. In addition, the SYNTAX III REVOLUTION trial demonstrated that CTCA combined with FFR-CT influenced heart team decision-making in patients with complex multivessel coronary artery disease [27]. Beyond lesion selection, FFR-CT has also enabled the development of virtual PCI planning tools. These platforms allow simulation of stent deployment by digitally modifying coronary lesions and recalculating FFR values, providing an estimate of post-procedural physiological outcomes before intervention [28]. Clinical studies have shown good agreement between predicted and measured post-PCI FFR values, supporting the feasibility of this approach [29]. From a PCI planning perspective, FFR-CT influences procedural decision-making beyond determining whether a lesion is ischemia-producing. A focal pressure gradient across a discrete stenosis may support localized PCI with limited stent implantation, whereas diffuse physiological decline without a clear focal gradient may suggest diffuse atherosclerotic disease with less expected benefit from focal PCI [20,21].

In multivessel disease, lesion-specific FFR-CT mapping may assist in prioritizing culprit lesions, determining completeness of revascularization, and avoiding unnecessary treatment of angiographically intermediate stenoses [21,26]. Virtual PCI simulation platforms further extend this concept by digitally modifying coronary lesions and predicting post-PCI physiological improvement before intervention [28].

Despite these advantages, FFR-CT remains associated with several limitations. Accurate computation depends on high-quality CT datasets and may be impaired by motion artefacts, severe calcification, prior coronary stents, obesity, and arrhythmias [22,26]. In addition, computational analysis may increase cost and delay workflow integration in some centers. The accuracy of FFR-CT in diffuse coronary disease and serial lesions also remains challenging because physiological pressure loss may be distributed across multiple segments rather than localized to a single stenosis.

Overall, FFR-CT represents a critical step toward non-invasive, physiology-guided PCI planning, bridging the gap between anatomical imaging and functional decision-making, and supporting more precise, efficient, and individualized PCI strategies.

*Case Example 1: Left Main Stem PCI Guided by CTCA and FFR-CT*

A 64-year-old male with hypertension and dyslipidemia presented with exertional angina despite optimal medical therapy. Baseline electrocardiography demonstrated ST-segment elevation in lead aVR with diffuse ST depression, a pattern suggestive of high-risk coronary anatomy, including possible left main stem or proximal multivessel disease. Coronary CT angiography (CTCA) revealed CTCA further demonstrated distal left main involvement extending into the LAD-LCx bifurcation with moderate eccentric calcification and a bifurcation angle of approximately 78°. The proximal left main reference diameter measured 4.8 mm, while proximal LAD and LCx diameters measured 3.7 mm and 3.4 mm respectively. Plaque distribution was predominantly located along the lateral wall extending toward the LAD ostium with relative sparing of the carina. These findings supported a provisional one-stent strategy from the left main into the LAD with jailed-wire protection of the LCx. CTCA-derived vessel sizing guided selection of a 4.0 mm drug-eluting stent followed by proximal optimization technique (POT). The absence of severe circumferential calcium or diffuse LCx ostial disease reduced the anticipated need for atherectomy or planned two-stent bifurcation PCI

### 2.2. Artificial Intelligence in the CTCA Workflow

Artificial intelligence (AI) is increasingly integrated into the CTCA workflow, transforming image acquisition, reconstruction and interpretation. Although coronary plaque quantification can be performed manually using conventional CT software, as image analysis was performed using software version 10.16SP0001E, artificial intelligence substantially improves automation, reproducibility, and workflow efficiency. AI-based algorithms can automatically segment coronary arteries, quantify plaque burden, characterize plaque composition, and identify adverse plaque features within minutes, thereby reducing analysis time and interobserver variability [30,31,32].

From a PCI planning perspective, AI integration enables rapid generation of clinically actionable information before intervention. Automated assessment of calcium arc, plaque length, vessel diameter, remodeling index, and low-attenuation plaque burden may help operators anticipate lesion complexity, determine the likelihood of stent underexpansion, and plan lesion-preparation strategies before entering the catheterization laboratory [31,33].

Beyond image reconstruction, AI has enabled automated coronary segmentation and plaque characterization, addressing the limitations of manual analysis, which is time-consuming and subject to interobserver variability [29,34]. Using convolutional neural networks trained on large datasets, AI can rapidly identify and quantify plaque components, including calcified and non-calcified plaque, with high reproducibility.

**Figure 2:**
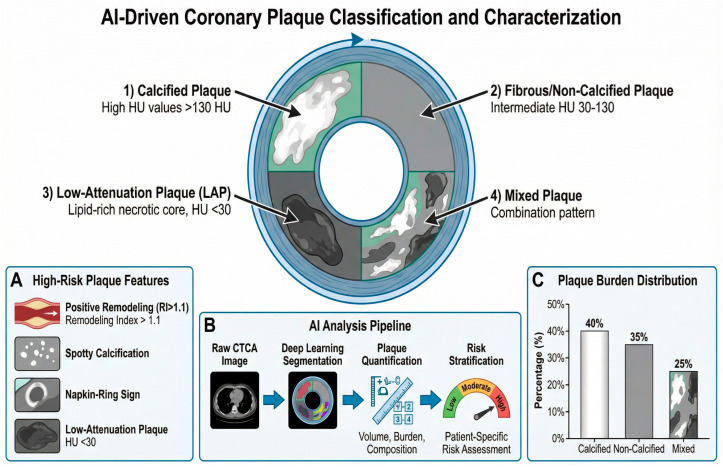
AI-driven coronary plaque classification and characterization, illustrating plaque subtypes and the AI analysis pipeline from raw image segmentation to patient-specific risk stratification. (**A**) High-risk plaque features on CTCA include (1) calcified plaque (HU > 130), (2) fibrous/non-calcified plaque (HU 30–130), (3) low-attenuation plaque (HU < 30), and (4) mixed plaque (combination pattern). (**B**) AI analysis pipeline showing steps from raw CTCA input through deep learning segmentation and feature extraction to high-risk plaque prediction. (**C**) Plaque burden distribution categorized as low (<25%), moderate (25–70%), or high (>70%).

From a clinical perspective, AI-driven plaque characterization provides valuable insight into lesion vulnerability. Features such as low-attenuation plaque, positive remodeling, spotty calcification, and the napkin-ring sign have been associated with increased risk of adverse cardiovascular events [31,35,36]. Automated detection of these features enables more comprehensive assessment of coronary disease beyond simple luminal narrowing. An overview of AI-driven coronary plaque analysis and its role in risk stratification and PCI planning is illustrated in Figure 2. Overall, AI represents a key component in the evolution of CTCA from a diagnostic imaging modality to an integrated platform for pre-procedural PCI planning, supporting more efficient, standardized, and personalized interventional care.

AI-assisted reporting represents an important step toward the clinical integration of CTCA into routine PCI planning. The concept of the ‘bionic radiologist’—a human expert augmented by AI tools—reflects a realistic model in which AI enhances, rather than replaces, clinical decision-making [32]. In the context of CTCA, AI-based platforms can streamline the reporting workflow by automatically performing coronary segmentation, stenosis grading, plaque quantification, and integration of FFR-CT values into structured reports. These systems reduce interobserver variability, improve efficiency, and enable faster generation of clinically relevant outputs, with some platforms completing full analyses within minutes [33]. From a PCI planning perspective, the value of AI-assisted reporting extends beyond efficiency to clinical applicability.

Structured CTCA reporting refers to standardized reporting systems integrating anatomical stenosis severity, plaque composition, calcium distribution, vessel dimensions, FFR-CT values, and procedural planning considerations into a unified PCI-oriented format. Rather than descriptive interpretation alone, structured reports generate clinically actionable outputs including lesion length measurements, reference vessel sizing, anticipated calcium-modification requirements, procedural complexity assessment, and suggested landing zones for PCI [14,37].

**Figure 3:**
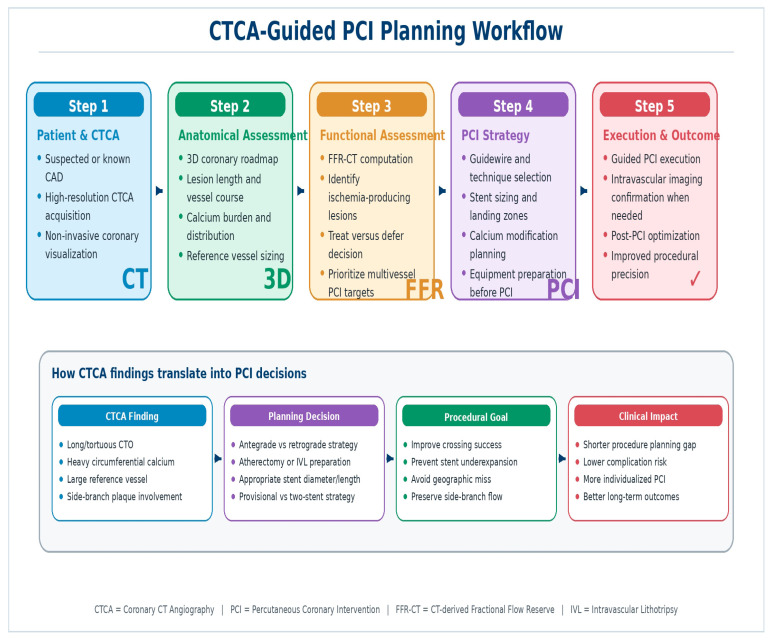
Schematic flowchart illustrating the comprehensive CTCA-guided PCI planning workflow, from initial patient presentation through integrated pre-procedural planning and post-procedural outcome optimization.

By integrating anatomical, functional, and plaque-level information into a single structured output, these systems facilitate the translation of CTCA findings into actionable procedural decisions, including lesion selection, stent sizing, calcium-modification planning, and procedural risk stratification before intervention. Overall, the integration of FFR-CT, AI-assisted plaque analysis, and structured CTCA reporting supports a more comprehensive pre-procedural planning pathway. This pathway links anatomical assessment, functional evaluation, lesion preparation, equipment selection, PCI execution, and post-PCI optimization within a single workflow. The integrated CTCA-guided PCI planning workflow is summarized in Figure 3.

Step 4 of the CTCA-guided PCI workflow represents the transition from image interpretation to procedural strategy formulation. CT-derived parameters directly influence PCI planning in several ways. Severe circumferential calcium (arc > 180°), calcium thickness > 0.5 mm, or lesion length > 5 mm may predict inadequate stent expansion and therefore support upfront plaque-modification strategies such as rotational atherectomy or intravascular lithotripsy [19,38]. Significant vessel tortuosity may prompt use of guide-extension catheters or more deliverable stent platforms, while bifurcation geometry and plaque distribution may influence selection between provisional stenting and planned two-stent techniques. CTCA-derived reference vessel diameters and lesion length measurements may additionally guide stent sizing and landing-zone selection before PCI [19,37].

### 2.3. Next-Generation CT Hardware (Second Comment)

Recent advances in CT hardware have further expanded the role of CTCA in PCI planning. Photon-counting detector CT provides substantially improved spatial resolution and contrast-to-noise performance compared with conventional energy-integrating detector systems [39]. These developments reduce calcium blooming artefacts and improve visualization of lumen boundaries in heavily calcified coronary arteries. From an interventional perspective, improved spatial resolution enables more accurate assessment of calcium arc, thickness, and longitudinal distribution, parameters that directly influence lesion preparation strategy and selection of calcium-modification devices. Spectral CT and material decomposition techniques may additionally improve differentiation between calcified, fibrotic, and lipid-rich plaque components.

Ultra-high-resolution scanners and motion-correction algorithms also improve visualization of distal vessel anatomy, bifurcation geometry, and chronic total occlusion morphology, potentially enhancing procedural planning in complex PCI scenarios [39].

## 3. Clinical Applications in Complex PCI

The clinical value of CTCA-guided PCI planning is most pronounced in anatomically complex scenarios, where the limitations of conventional angiography are most consequential and where pre-procedural anatomical and functional information can directly influence procedural strategy and outcomes. This section examines the role of CTCA in key PCI planning domains, including chronic total occlusion PCI, left main and bifurcation intervention, stent optimization, vessel sizing, and lesion preparation. The following case demonstrates how CTCA-derived assessment of coronary calcification can directly influence lesion preparation strategy and device selection during complex PCI.

*Case Example 2: IVL-Assisted PCI for Severe Calcific Proximal LAD Disease*

A patient presented with exertional angina despite optimal medical therapy. CTCA demonstrated severe concentric calcification involving the proximal LAD with calcium arc approximately 270°, calcium thickness exceeding 0.7 mm, and longitudinal calcium extension measuring approximately 12 mm. Peak calcium attenuation exceeded 950 HU. Reference vessel diameter measured 3.5 mm proximally and 3.0 mm distally. These findings predicted reduced lesion compliance and high risk of stent underexpansion.

**Figure 4:**
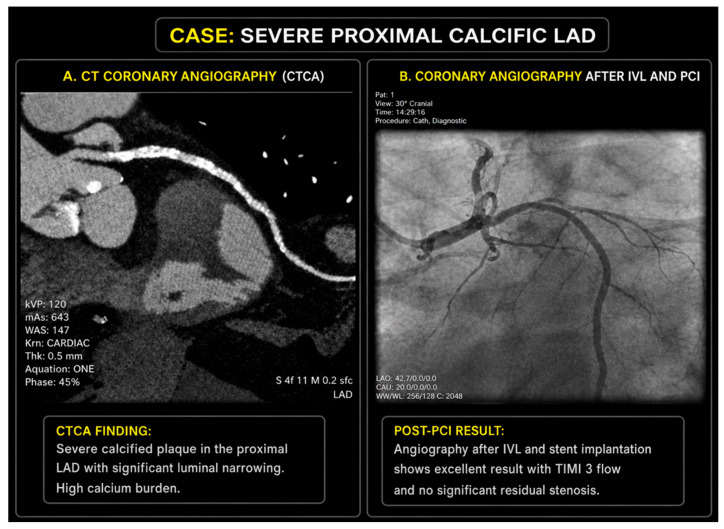
CTCA-guided lesion preparation in severe calcified proximal LAD disease. (**A**) CTCA demonstrating severe concentric calcification of the proximal LAD with an estimated calcium arc > 270°, calcium thickness > 0.7 mm, lesion length approximately 24 mm, and high-attenuation plaque (>900 HU), indicating high risk of stent underexpansion. (**B**) Final coronary angiography following intravascular lithotripsy-assisted PCI and drug-eluting stent implantation demonstrating optimal stent expansion and TIMI 3 flow.

Because the lesion demonstrated deep circumferential calcium rather than focal superficial nodular calcium, intravascular lithotripsy (IVL) was selected as the preferred calcium-modification strategy prior to stent implantation [19,38]. Coronary angiography confirmed severe calcific proximal LAD disease. IVL was successfully performed followed by implantation of a drug-eluting stent with excellent angiographic expansion and TIMI 3 flow (Figure 4).

### 3.1. CTCA-Guided Lesion Preparation and PCI Optimization

In selected calcified lesions, CTCA can help define calcium burden and lesion morphology before PCI, supporting appropriate lesion preparation and device selection. The following case illustrates CTCA-guided planning for calcified mid-RCA stenosis treated with drug-eluting balloon angioplasty.

*Case Example 3: CTCA-Guided PCI with DEB Angioplasty for Calcified Mid-RCA Stenosis*

**Figure 5:**
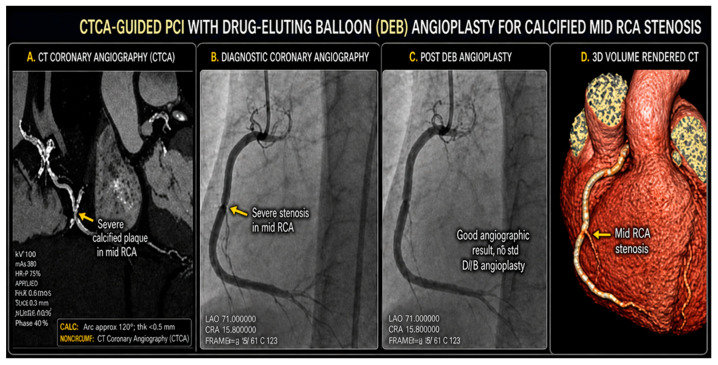
CTCA-guided PCI with Drug Eluting Balloon (DEB) angioplasty for calcified mid-RCA stenosis. (**A**) Curved multiplanar CTCA reconstruction demonstrating eccentric calcified plaque involving the mid RCA with calcium arc approximately 120°, calcium thickness < 0.5 mm, and lesion length approximately 18 mm, consistent with non-circumferential superficial calcification suitable for balloon-based lesion preparation. (**B**) Diagnostic coronary angiography confirming calcific mid-RCA stenosis. (**C**) Final angiographic result following lesion preparation and drug-coated balloon (DCB) angioplasty demonstrating good distal flow with no significant residual stenosis. (**D**) Three-dimensional volume-rendered CT reconstruction illustrating lesion location, vessel course, and spatial distribution of calcification within the mid RCA.

A male patient in his late 60s presented with exertional angina despite optimal medical therapy. CTCA demonstrated focal eccentric calcification involving the mid RCA with calcium arc approximately 120°, calcium thickness < 0.5 mm, and lesion length approximately 18 mm. Peak calcium attenuation measured approximately 650 HU. Reference vessel diameter was preserved without diffuse disease or severe concentric calcification (Figure 5).

Because the lesion demonstrated focal calcification with preserved vessel compliance and absence of extensive circumferential calcium burden, lesion preparation using specialty balloons followed by drug-eluting balloon angioplasty was considered feasible without atherectomy or stent implantation [19,38]. Final angiography demonstrated an excellent procedural result with TIMI 3 flow and no significant residual stenosis.

#### 3.1.1. Precise Vessel Sizing and Stent Optimization

CTCA-derived three-dimensional reconstruction enables accurate measurement of proximal and distal reference vessel diameters, lesion length, and landing-zone anatomy before PCI [19,37]. These measurements may improve pre-procedural stent sizing, reduce geographic miss, and minimize risk of stent malapposition or underexpansion. In long or tapered lesions, CTCA may additionally identify vessel diameter mismatch between proximal and distal reference segments, thereby facilitating selection of appropriate stent diameter and optimization strategies such as proximal optimization technique (POT).

**Figure 6:**
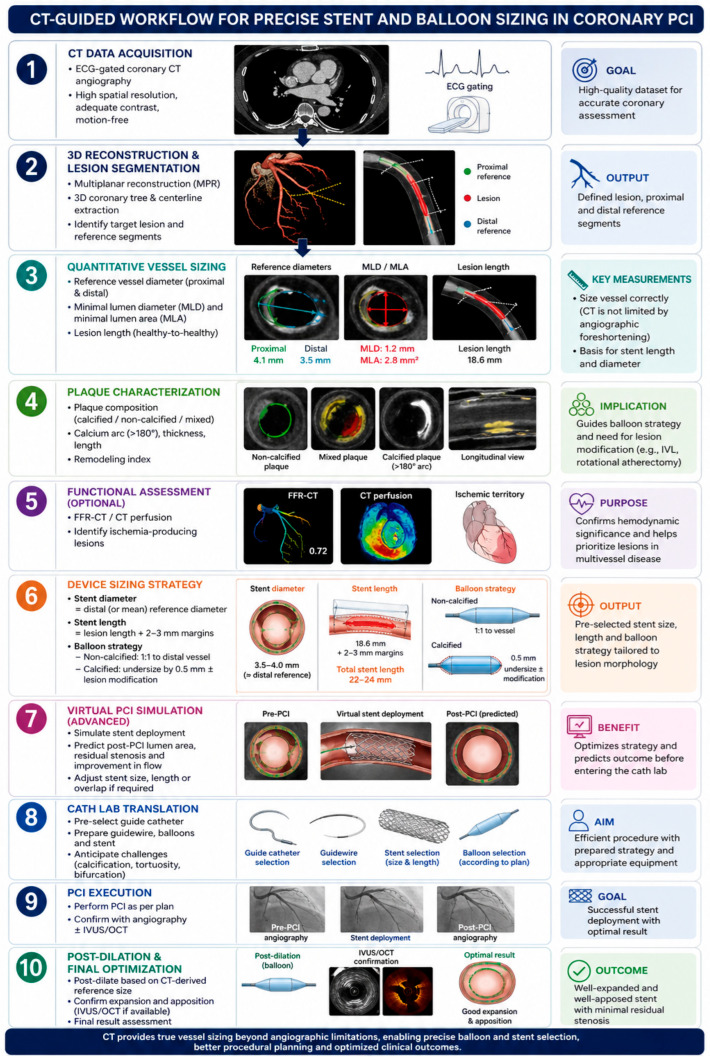
A comprehensive CT-guided workflow for precise balloon and stent sizing in coronary PCI, organized as a stepwise, visually structured algorithm from image acquisition to final procedural optimization.

The procedural workflow illustrated in Figure 6 is reflected in Cases 2–4, where CT-derived measurements of calcium burden, lesion length, vessel dimensions, and plaque morphology directly influenced lesion-preparation strategy, device selection, and PCI execution.

#### 3.1.2. CTCA-Guided Calcium Modification Planning

CTCA may also improve lesion preparation strategy by defining the extent and pattern of coronary calcification before PCI.

**Figure 7:**
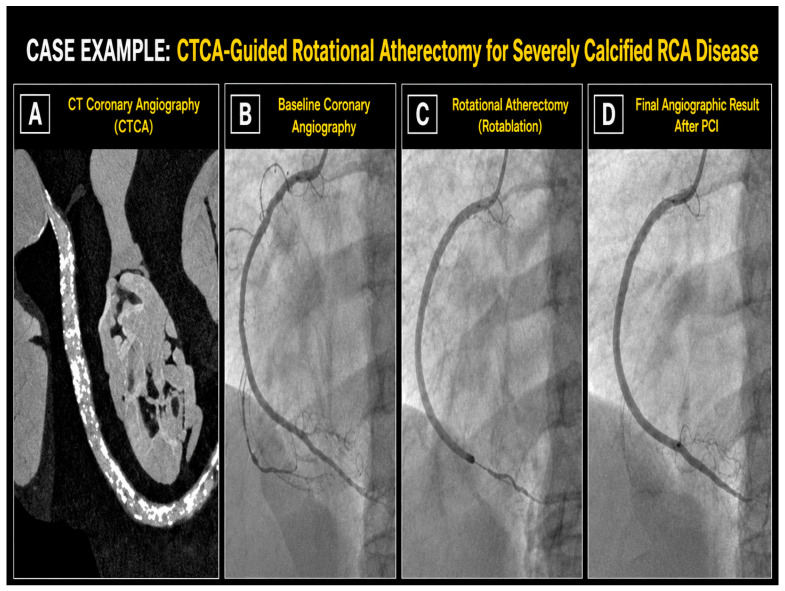
CTCA-guided rotablation for severely calcified RCA disease. (**A**) CTCA demonstrating diffuse circumferential calcification involving the proximal-to-mid RCA with calcium arc > 180°, longitudinal calcium extension > 5 mm, and high-attenuation plaque (>900 HU), indicating high risk of balloon failure and stent underexpansion. (**B**) Diagnostic coronary angiography confirming severe calcific RCA stenosis. (**C**) Rotational atherectomy performed for calcium modification before stent implantation. (**D**) Final angiographic result demonstrating optimal stent expansion with TIMI 3 distal flow and no significant residual stenosis.

Knowledge of calcium arc, depth, and longitudinal distribution can help operators anticipate whether plaque modification will be required and select among techniques such as rotational atherectomy, orbital atherectomy, cutting or scoring balloons, or intravascular lithotripsy [38]. This allows a more deliberate strategy rather than reactive intraprocedural decision-making. A representative case demonstrating CTCA-guided selection of rotational atherectomy for severely calcified RCA disease is shown in Figure 7.

*Case Example 4: CTCA-Guided Rotablation for Severely Calcified RCA Disease*

A 67-year-old male with multiple cardiovascular risk factors presented with exertional angina despite optimal medical therapy. Coronary CT angiography demonstrated severe diffuse calcification of the right coronary artery (RCA), with extensive calcified plaque involving the proximal-to-mid RCA and associated luminal narrowing.

Given the heavy calcium burden identified on CTCA, the lesion was considered at high risk for inadequate balloon expansion and stent underexpansion. Therefore, CTCA findings directly guided the procedural strategy, supporting the planned use of rotational atherectomy for calcium modification before stent implantation.

Invasive coronary angiography confirmed a severely calcified RCA lesion. Rotablation was performed successfully, followed by balloon preparation and drug-eluting stent implantation. Final angiography demonstrated an optimal result with good distal flow and no immediate complications.

This case illustrates the value of CTCA in identifying severe coronary calcification before PCI and guiding lesion preparation strategy, particularly the selection of rotational atherectomy in complex calcified RCA disease.

Specific CT-derived calcium parameters may help guide selection of plaque-modification technologies. Circumferential calcium with arc > 180°, thickness > 0.5 mm, and longitudinal extension > 5 mm predicts increased risk of stent underexpansion and may support upfront calcium-modification strategies [19,38]. Deep concentric calcium may favor intravascular lithotripsy because acoustic pressure waves can fracture deep calcium layers, whereas superficial protruding calcium or calcified nodules may be more effectively modified using rotational or orbital atherectomy. Focal eccentric calcification with preserved vessel compliance may be adequately treated using scoring or cutting balloons alone.

#### 3.1.3. Multi-Vessel Disease and Revascularization Completeness

In multivessel coronary disease, combining CTCA with FFR-CT offers a framework that integrates anatomical stenosis burden with lesion-specific physiological relevance, which may support better-informed decisions about the extent and completeness of revascularization [40].

### 3.2. Chronic Total Occlusion PCI: CTCA-Guided Procedural Planning

Chronic total occlusions (CTOs) represent one of the most technically challenging subsets of percutaneous coronary intervention, with procedural success highly dependent on accurate pre-procedural planning. The complexity of CTO lesions arises from factors such as occlusion length, proximal cap morphology, vessel tortuosity, calcification, and the quality of distal vessel visualization, all of which may be incompletely assessed by invasive angiography alone [41,42]. CTCA provides a unique advantage in CTO PCI by offering a three-dimensional visualization of the occluded vessel, enabling detailed assessment of lesion characteristics before intervention. Key parameters that can be evaluated include occlusion length, vessel course within the occluded segment, proximal and distal cap morphology, and the extent and distribution of calcification [43]. The CCTA-based J-CTO score (which includes five parameters: lesion length > 20 mm, calcification, bending > 45°, proximal cap ambiguity, and previously failed attempts) had superior performance than the invasive angiography-based J-CTO score in one study [44]. Later, the CT-RECTOR multicenter registry was the first to derive a score from pre-procedural CCTA in 240 patients with CTO lesions. The CT-RECTOR score assigns 1 point to each of the following variables: multiple occlusions, blunt cap shape at the entry or exit site of the occlusion, severe calcification, bending > 45°, duration > 12 months or unknown, and reattempt procedure. The authors classified the CTO lesions as easy (score 0), intermediate (score 1), difficult (score 2) and very difficult (score ≥ 3). The four groups were associated with successful lesions crossing within less than 30 min in 95%, 88%, 57% and 22% of the cases, respectively. Similarly, the rates of final procedural success were 95%, 91%, 66% and 40%, respectively. The authors concluded that the CCTA-derived score showed a higher discrimination than the J-CTO score [45]. The KCCT score assigns 1 point to each of the following variables: proximal blunt cap shape, proximal adjacent side branch, occlusion length ≥ 15 mm, bending > 45°, duration > 12 months or unknown, reattempt procedure, and severe peripheral calcification; 2 points are assigned to central calcification [46]. From a practical standpoint, CTCA findings can be directly translated into procedural strategy. Proximal cap ambiguity identified on CTCA may prompt consideration of alternative crossing strategies, such as a retrograde approach or dissection-and-re-entry techniques. Conversely, a short, tapered occlusion with minimal calcification may support an antegrade wire escalation strategy.

In addition, CTCA can assist in guidewire selection by providing information on vessel tortuosity and lesion composition. Severe calcification or long occlusion segments may require specialized CTO wires, microcatheters and calcium modification strategies, while visualization of the distal vessel can improve targeting accuracy and reduce procedural uncertainty. Additionally, CCTA identifies lesion composition, distinguishing between heavily calcified, fibrotic, and lipid-rich segments. Calcified CTO may require specialized equipment like atherectomy or intravascular lithotripsy, while less calcified lesions might be approachable with conventional balloons [47].

**Figure 8:**
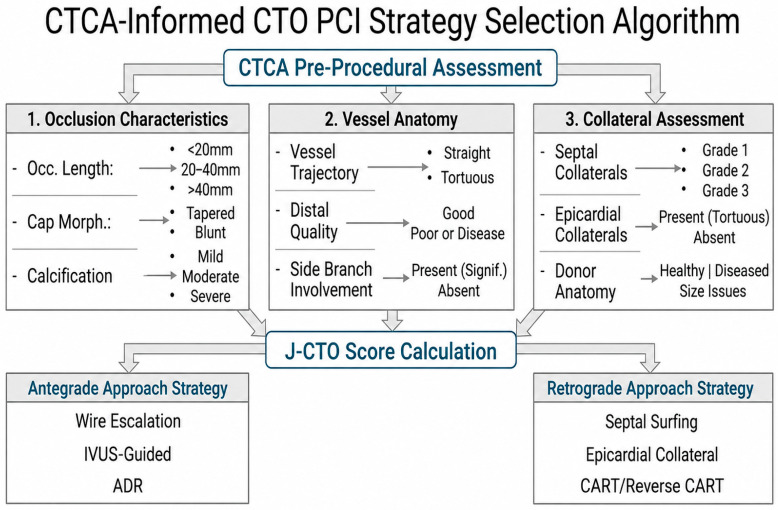
Algorithmic framework for CTCA-informed CTO PCI strategy selection, integrating anatomical parameters to guide the selection between antegrade and retrograde crossing strategies.

The clinical relevance of CTCA-guided CTO planning has been demonstrated in randomized and observational studies. The CT-CTO trial showed that pre-procedural CTCA significantly improved procedural success rates compared with angiography alone, while also reducing procedure time, contrast volume, and radiation exposure [45,48]. Overall, CTCA-guided planning in CTO PCI enables a shift from reactive intra-procedural decision-making to proactive strategy formulation, improving procedural efficiency, success rates, and operator confidence (Figure 8).

In practical application, CTCA-derived CTO assessment may guide selection between antegrade and retrograde crossing strategies before intervention begins. Short occlusions with tapered proximal caps, limited calcification, and minimal tortuosity are generally favorable for antegrade wire escalation. In contrast, ambiguous proximal caps, severe calcification, long occlusion length (>20 mm), or vessel bending > 45° increase procedural complexity and may support early consideration of retrograde crossing or dissection–reentry techniques [44,45,46]. Visualization of distal vessel quality and collateral anatomy on CTCA may additionally influence guidewire selection, microcatheter support, and procedural sequencing.

### 3.3. Left Main and Bifurcation PCI: Three-Dimensional Planning for Two-Dimensional Complexity

Left main coronary artery disease and bifurcation lesions represent some of the most technically demanding PCI scenarios, where inadequate procedural planning may lead to suboptimal outcomes.

Conventional angiography, as a two-dimensional luminographic technique, may underestimate lesion complexity, plaque distribution, and vessel geometry in these settings. CTCA provides a comprehensive three-dimensional assessment that is particularly valuable in left main and bifurcation PCI planning. Key parameters include accurate measurement of vessel diameter, lesion length, bifurcation angle, and plaque distribution across both the main vessel and side branch [49]. From a procedural standpoint, CTCA findings can be directly translated into interventional strategy. In left main disease, precise measurement of ostial and shaft dimensions supports appropriate stent sizing and optimal landing-zone selection.

In bifurcation lesions, detailed assessment of plaque distribution and side-branch involvement can guide the choice between provisional stenting and planned two-stent techniques. In addition, CTCA enables accurate evaluation of calcium burden and distribution, which may influence lesion preparation strategies such as rotational atherectomy or intravascular lithotripsy. Assessment of vessel geometry and bifurcation angle can further support selection of stent technique and procedural optimization. The clinical relevance of CTCA in this context is supported by the SYNTAX III REVOLUTION trial, which demonstrated that CTCA combined with FFR-CT significantly influenced heart team decision-making in patients with complex multivessel and left main coronary artery disease [27]. Detailed CTCA analysis may substantially influence bifurcation PCI strategy selection. Wide bifurcation angles, extensive side-branch ostial disease, large side-branch diameter, and diffuse plaque extending into both daughter vessels may support planned two-stent strategies such as DK-crush or culotte techniques [18,49]. In contrast, focal plaque confined predominantly to the main vessel with minimal side-branch involvement may favor provisional stenting. CTCA assessment of plaque distribution relative to the carina may additionally help predict the likelihood of side-branch compromise after main-vessel stenting. In heavily calcified left main bifurcation lesions, circumferential ostial calcium and marked vessel diameter mismatch may further support pre-procedural plaque-modification strategies before stent implantation. Overall, CTCA-guided planning in left main and bifurcation PCI enhances anatomical understanding and supports more informed procedural decision-making, contributing to improved procedural outcomes.

## 4. Comparative Analysis: CTCA Versus Invasive Coronary Angiography in PCI Planning

The fundamental differences between coronary CT angiography (CTCA) and invasive coronary angiography (ICA) as pre-procedural planning tools are substantial and clinically significant. While ICA remains the reference standard for real-time luminal assessment, it is inherently limited by its two-dimensional nature and inability to characterize plaque or assess vessel wall morphology. In contrast, CTCA provides a comprehensive three-dimensional evaluation of coronary anatomy, enabling detailed assessment of plaque composition, vessel geometry, and lesion characteristics prior to intervention. In addition, CTCA allows integration with functional assessment through FFR-CT, providing a combined anatomical and physiological framework for clinical decision-making. A comparison between CTCA and ICA in the context of PCI planning is summarized in Table 1.

## 5. Current Challenges and Emerging Solutions

Despite the compelling evidence base and technological advances described above, the widespread adoption of CTCA-guided PCI planning faces several practical and technical challenges. These challenges relate to image quality, workflow integration, computational demands, operator familiarity, and accessibility. Dense coronary calcification may cause blooming artefacts, motion artefacts can reduce diagnostic confidence, and FFR-CT computation may remain challenging in some workflows. In addition, limited integration of CTCA datasets into catheterization laboratories may delay adoption in routine PCI planning. The principal challenges and emerging solutions for CTCA-guided PCI planning are summarized in Table 2.

## 6. Future Perspectives: Toward a New Era of Precision Interventional Cardiology

Advances in CT technology, computational modeling, and artificial intelligence are expected to further expand the role of CTCA in interventional cardiology, moving toward a more integrated and precision-guided approach to PCI planning.

### 6.1. Photon-Counting Detector CT: The Next Hardware Revolution

Photon-counting detector CT represents an important hardware development that may further improve coronary imaging. Its advantages include higher spatial resolution and spectral capabilities, which may reduce calcium blooming artefacts and enhance plaque characterization through advanced material decomposition techniques.

### 6.2. Digital Twin Technology and Patient-Specific Simulation

Another emerging concept is the development of patient-specific digital twins that model coronary anatomy and physiology in a dynamic and interactive manner. These systems may enable simulation of alternative revascularization strategies and support more individualized and predictive procedural planning.

### 6.3. Integration with Hybrid Catheterization Laboratories

Hybrid catheterization laboratories may provide a future environment in which CTCA data are directly integrated with live PCI guidance. Co-registration of CT-derived anatomical information with fluoroscopy could enable a more seamless workflow, allowing imaging and intervention to converge within a unified procedural platform.

## 7. Discussion

This review highlights the evolving role of coronary CT angiography (CTCA) as a comprehensive pre-procedural planning tool in percutaneous coronary intervention (PCI). Traditionally, invasive coronary angiography (ICA) has served as the reference standard for coronary assessment; however, its inherent limitation as a two-dimensional luminographic technique restricts its ability to fully characterize vessel morphology, plaque composition, and lesion complexity. The integration of CTCA into PCI planning represents a paradigm shift from reactive, intra-procedural decision-making to a more proactive and data-driven strategy. By providing three-dimensional anatomical detail, CTCA enables accurate assessment of lesion length, vessel diameter, plaque characteristics, and spatial relationships within the coronary tree. When combined with functional assessment through FFR-CT and advanced plaque analysis, CTCA offers a comprehensive framework that integrates both anatomical and physiological information. The clinical applications discussed in this review demonstrate the value of CTCA in complex PCI scenarios. In chronic total occlusions, CTCA improves procedural planning by clarifying lesion morphology and guiding crossing strategies. In left main and bifurcation disease, it supports precise stent sizing, strategy selection, and procedural optimization.

In addition, CTCA contributes to improved lesion preparation through accurate characterization of calcification and vessel geometry. Despite these advantages, several challenges remain. Image quality may be affected by motion artefacts and heavy calcification, and integration of CTCA data into catheterization laboratory workflows is not yet universally established. Furthermore, the availability and cost of advanced techniques such as FFR-CT may limit widespread adoption in certain healthcare settings. Future developments are expected to address many of these limitations. Advances in CT hardware, artificial intelligence, and computational modeling may enhance image quality, reduce analysis time, and facilitate real-time integration of CTCA into interventional workflows. Emerging concepts such as digital twin technology and hybrid imaging environments may further enable personalized and predictive PCI planning. Recent expert consensus from the SCAI/SCCT roundtable further supports the use of CTCA to guide PCI planning in selected clinical scenarios [37].

This reinforces the growing view that CTCA should be considered not only as a diagnostic test, but also as a practical tool for procedural preparation, lesion selection, and strategy optimization. Overall, CTCA-guided PCI planning represents a significant advancement in interventional cardiology, offering the potential to improve procedural precision, optimize clinical outcomes, and support a more individualized approach to coronary revascularization.

## 8. Conclusions

Coronary computed tomography angiography has undergone a fundamental transformation in its clinical role, evolving from a tool for the non-invasive exclusion of obstructive CAD into a comprehensive pre-procedural planning platform for percutaneous coronary intervention. This evolution has been driven by three converging technological advances—FFR-CT, AI-assisted plaque characterization, and next-generation CT hardware—and validated by an increasingly robust body of clinical trial evidence. The key messages from the evidence reviewed here are clear. First, CTCA-guided diagnostic strategies are associated with improved long-term cardiovascular outcomes, as demonstrated by the SCOT-HEART 10-year data [5]. Second, FFR-CT reduces unnecessary invasive angiography and improves heart team decision-making in complex CAD, as shown by the PLATFORM and SYNTAX III REVOLUTION trials [27]. Third, pre-procedural CTCA improves procedural success in CTO-PCI, as demonstrated by the CT-CTO trial. Fourth, ongoing trials including PRECISION-CT are poised to provide definitive evidence for CTCA-guided PCI as a standard of care. Challenges remain—particularly regarding calcification artefact, workflow integration, and cost—but these are being progressively addressed through technological innovation and expanding clinical experience. As photon-counting CT, near-real-time FFR-CT computation, and AI-assisted analysis become more widely available, the barriers to routine CTCA-guided PCI planning will continue to diminish. The field of interventional cardiology is at a clear inflection point. The question is no longer whether CTCA can improve PCI planning—it can. The question is how quickly and how systematically this capability can be integrated into routine clinical practice for the benefit of patients.

## Figures and Tables

**Table 1:** Comparative analysis of key attributes of invasive coronary angiography (ICA) versus coronary CT angiography (CTCA) as pre-procedural planning tools for PCI.

Parameter	Invasive Coronary Angiography (ICA)	Coronary CT Angiography (CTCA)
Dimensionality	2D luminography only	True 3D isotropic reconstruction
Plaque Assessment	Luminogram only; no plaque characterization	Full plaque characterization (calcified, non-calcified, LAP)
Functional Data	Requires separate pressure wire (invasive FFR)	Non-invasive FFR-CT from resting scan
CTO Visualization	Cannot visualize distal vessel	Complete vessel course mapping
Invasiveness	Invasive; vascular access required	Non-invasive; no vascular access
Radiation Exposure	Moderate (5–15 mSv)	Low–moderate (2–5 mSv with modern protocols)
Contrast Volume	Moderate–high (50–150 mL)	Low–moderate (50–80 mL)
Procedural Planning	Reactive, intra-procedural decision-making	Proactive, pre-procedural strategy formulation
Stent Sizing	Qualitative, often requires IVUS/OCT	Quantitative 3D measurement
Calcium Characterization	Limited (qualitative)	Detailed (quantitative, arc, depth, distribution)

**Table 2:** The summary of CT-derived parameters which have direct procedural implications during PCI planning. Rather than serving purely descriptive purposes, these measurements may guide lesion-preparation strategy, device selection, procedural sequencing, prediction of stent expansion, and estimation of technical complexity before intervention is undertaken [18,19,20,21,22,23,24,25,26,27,28,29,30,31,32,33,34,35,36,37,38,39,40,41,42,43,44].

CT Parameters	Definition/Assessment Method (CTCA/FFR-CT)	Interventional Implication	Impact on Equipment Selection/Technique
Calcium Burden (Agatston/segmental CAC)	Quantification of calcium score; segmental visual grading	Predicts lesion rigidity and stent underexpansion risk	High CAC → rotational atherectomy, orbital atherectomy, or IVL; adjunctive NC balloons
Calcium Distribution Pattern	Arc (°), length, superficial vs. deep calcium	Circumferential (>180°) calcium predicts poor expansion	Diffuse/circumferential → atherectomy or IVL; focal → scoring/cutting balloons
Calcium Thickness	Measured on cross-sectional CT	Thickness > 0.5 mm predicts balloon resistance	Favor IVL for deep thick calcium
Lesion Length	Centerline measurement	Determines stent coverage strategy	Long lesions → long/overlapping DES; consider deliverability strategies
Reference Vessel Diameter (RVD)	Proximal/distal lumen sizing	Accurate stent sizing, reduces geographic miss	Direct guidance for stent diameter selection (CT-based sizing improves outcomes [29])
Minimal Luminal Area (MLA)	Smallest lumen cross-sectional area	Correlates with ischemia	Supports PCI indication, especially with FFR-CT integration
Plaque Composition	Calcified, fibrous, lipid-rich, mixed	Lipid-rich plaques → embolic risk	Gentle pre-dilation; avoid aggressive early expansion
Vessel Tortuosity	Curvature along vessel path	Impacts device deliverability	Use guide extension, supportive wires, flexible DES platforms
Bifurcation Anatomy	SB angle, size, plaque distribution	Determines PCI strategy	Large SB + wide angle → planned two-stent techniques (DK-crush, culotte)
CTO Characteristics	Cap morphology, length, calcification	Predicts procedural complexity	Ambiguous cap → retrograde approach; calcified → plaque modification
Virtual PCI Simulation (FFR-CT)	Predicted post-PCI physiology	Estimates benefit of intervention	Helps avoid unnecessary stenting; optimizes lesion selection

## Data Availability

The original contributions presented in this study are included in the article. Further inquiries can be directed to the corresponding author.
